# Complete Circular Genome Sequences of Brachyspira hyodysenteriae Isolates of the Four Different Sequence Types Causing Swine Dysentery in Switzerland

**DOI:** 10.1128/MRA.00847-21

**Published:** 2021-09-30

**Authors:** Ana B. García-Martín, Sarah Schmitt, Friederike Zeeh, Vincent Perreten

**Affiliations:** a Division of Molecular Epidemiology and Infectious Diseases, Institute of Veterinary Bacteriology, Vetsuisse Faculty, University of Bern, Bern, Switzerland; b Graduate School for Cellular and Biomedical Sciences, University of Bern, Bern, Switzerland; c Institute for Food Safety and Hygiene, Section of Veterinary Bacteriology, Vetsuisse Faculty, University of Zurich, Zurich, Switzerland; d Clinic for Swine, Department of Clinical Veterinary Medicine, Vetsuisse Faculty, University of Bern, Bern, Switzerland; University of Rochester School of Medicine and Dentistry

## Abstract

The complete genomes of four Brachyspira hyodysenteriae isolates of the four different sequence types (STs) (ST6, ST66, ST196, and ST197) causing swine dysentery in Switzerland were generated by whole-genome sequencing and *de novo* hybrid assembly of reads obtained from second (Illumina) and third (Oxford Nanopore Technologies and Pacific Biosciences) generation high-throughput sequencing.

## ANNOUNCEMENT

Brachyspira hyodysenteriae was confirmed to cause swine dysentery in Swiss pig herds in 2008 (1). Since then, isolates have been characterized by multilocus sequence typing and determination of their antimicrobial resistance mechanisms (2, 3). To date, only four sequence types (STs) (ST6, ST66, ST196, and ST197) of B. hyodysenteriae have been identified, prompting us to sequence their complete genomes.

Isolates obtained from our cryopreserved collections in Zurich (BHZ755 [ST6], BHZ375 [ST66], and BHZ526 [ST197]) and Bern (Bh743-7 [ST196]) were grown anaerobically at 42°C for 5 days on Trypticase soy agar with 5% (vol/vol) defibrinated sheep blood (Becton, Dickinson). The bacterial lawn of two plates was collected using a 10-μl plastic loop and resuspended in buffer (0.1 M Tris-HCl, 0.01 M NaCl, 0.1 M EDTA). Genomic DNA was extracted using the DNeasy blood and tissue kit (Qiagen), treated with RNase (20 mg/ml) at 37°C for 30 min, purified with AMPure XP magnetic beads (Beckman Coulter), and quantified with a Qubit 3.0 fluorometer (Life Technologies). Prior to Oxford Nanopore Technologies (ONT) long-read library preparation, DNA was sheared up to 20 kb using Covaris g-TUBEs. DNA libraries were obtained using the 1D native barcoding (EXP-NBD104) and ligation (SQK-LSK109) kits and loaded onto a FLO-MIN106D flow cell R9.4.1. Real-time sequencing was performed using a MinION Mk1B device (ONT), and results were visualized using the controlling software MINKNOW-GUI v19.05.0 (ONT). Base calling and demultiplexing were performed using Guppy v2.3.7 (ONT). Long reads were analyzed using NanoPack (4) and trimmed by 100 bp using Cutadapt v1.18 (5). Default parameters were used for all software unless otherwise specified.

Pacific Biosciences (PacBio) long-read libraries were prepared from DNA extracted as described previously (6), following the BluePippin ≧7-kb size selection method (with an insert length of 10 kb), and sequenced in a single-molecule real-time (SMRT) Cell v2 compatible with the Sequel II system at Lausanne Genomic Technologies Facility (Lausanne, Switzerland). Reads were demultiplexed using lima from the SMRT Analysis software (PacBioSuite-6.0.0.47841) (https://www.pacb.com/support/software-downloads) and analyzed using LongQC (7).

Short-read sequencing was performed using a NEBNext Ultra II directional DNA library with TruSeq adapters on an Illumina NovaSeq 6000 platform at Eurofins (Germany). Paired-end 2 × 150-bp reads were quality checked using FastQC v0.11.7 (8) and filtered with Trimmomatic v0.36 (9). ONT and Illumina libraries were prepared from the same DNA samples, and PacBio libraries were prepared from different batches of DNA obtained using the same culture conditions.

*De novo* hybrid assemblies were generated by running the -bold option of Unicycler v0.4.4 (10). Assembly polishing, circularization, and rotation were performed using Unicycler. Polished circular assemblies were visualized using Bandage v0.8.1 (11). Assembly quality was analyzed using QUAST v4.6.0 (12). The mean depth of coverage (Table 1) for the entire assembly was calculated by remapping Illumina reads by running the plugin BBMap v37.25 (13) in Geneious R10.2.3 (Biomatter, Ltd.). Additional comparisons of the hybrid ONT-Illumina assemblies with their PacBio counterparts allowed the detection of chromosomal low-complexity regions, which were corrected by Sanger sequencing (Table 1). Sanger sequencing samples were obtained from the same DNA as the ONT and Illumina libraries and were sequenced in both directions. Sequence chromatograms were visualized in Geneious to assess their quality. Untrimmed sequences were aligned to their corresponding assemblies, which were manually edited in Geneious. Assemblies consisted of two circular replicons (both characterized by low G+C contents of <27.1%), representing one chromosome and one plasmid, with mean lengths of 3,017,459 bp and 32,669 bp, respectively. Annotation was performed using the NCBI Prokaryotic Genome Annotation Pipeline (PGAP) v4.10 (14). On average, 2,576 protein-coding genes, 40 rRNAs, and 30 pseudogenes were identified on the chromosome (Table 1). Thirty protein-coding genes were identified in all plasmids except pBHZ375, which has 29 protein-coding genes.

**TABLE 1:** Metrics of the announced complete genomes of Brachyspira hyodysenteriae isolates

Parameter	Data for isolate:					
	Bh743-7	BHZ375	BHZ526		BHZ755	
Isolate information						
Isolation date	2017	2013	2014		2015	
ST	196	66	197		6	
Raw sequencing data						
ONT^*a*^						
No. of sequenced reads (Unicycler-loaded reads)	346,463	220,134	313,725		276,045	
Sequenced length (bp)	1,744,406,639	819,961,706	1,859,824,743		1,504,066,585	
Mean read length (bp)	5,235	3,925	6,128		5,649	
Read *N*_50_ (bp)	7,705	6,528	8,858		8,264	
PacBio^*b*^						
No. of sequenced reads	189,845	177,937	170,348		186,892	
No. of Unicycler-loaded reads	189,444	177,933	170,328		186,839	
Sequenced length (bp)	NA	NA	NA		NA	
Mean read length (bp)	7,037	7,229	6,940		6,984	
Read *N*_50_ (bp)	9,048	9,163	9,056		8,898	
Illumina^*c*^						
No. of sequenced reads (× 10^6^)	7.4	10.8	6.3		6.2	
Sequenced length (Mbp)	2,225	3,243	1,879		1,870	
Assembly^*d*^						
ONT-Illumina						
Total genome length (bp)	3,085,162	3,043,241	3,036,451		3,035,657	
Chromosome length (bp)	3,052,634	3,010,106	3,003,989		3,003,105	
Plasmid length (bp)	32,528	33,135	32,462		32,552	
G+C content (%)	27.0	27.0	27.1		27.1	
Coverage depth (mean ± SD) (×)						
Chromosome	691 ± 84	1,001 ± 119	590 ± 51		592 ± 56	
Plasmid	1,203 ± 118	3,510 ± 319	1,004 ± 97		1,036 ± 101	
PacBio-Illumina						
Total genome length (bp)	3,085,111	3,043,198	3,035,137		3,035,627	
Chromosome length (bp)	3,052,583	3,010,063	3,002,675		3,003,075	
Plasmid length (bp)	32,528	33,135	32,462		32,552	
G+C content (%)	27.1	27.0	27.1		27.1	
Coverage depth (mean ± SD) (×)						
Chromosome	691 ± 84	1,001 ± 119	590 ± 53		592 ± 56	
Plasmid	1,203 ± 118	3,510 ± 319	1,004 ± 97		1,036 ± 101	
ONT-Illumina hybrid assembly correction^*e*^						
No. of Sanger sequencing-corrected regions	3	1	1		2	
Total no. of nucleotides added per chromosome	59	15	9		27	
Primer sequences (5′ to 3′)						
Chromosomal region 1 (16 bp) (nucleotide positions 444843 to 444858)	Bh743-7-1F, AGTACCTTTTCCAGCAGCAAG; Bh743-7-1R, GCAGAGGTGAAGCCGCTAAA					
Chromosomal region 2 (7 bp) (nucleotide positions 449527 to 449533)	Bh743-7-2F, TGTACAAAATTATATGCCGCCATAA; Bh743-7-2R, TTGATGAGCGTATGTGGGAAT					
Chromosomal region 3 (36 bp) (nucleotide positions 2726169 to 2726204)	Bh743-7-3F, GGAAATAGTTGGGGAGAGGTTCA; Bh743-7-3R, TGGAAATAGTAAACCCAAATCTGTTG					
Chromosomal region 1 (15 bp) (nucleotide positions 1728861 to 1728875)	BHZ375-2F, CCCCATAAAAAGCTTTGAATCCA; BHZ375-2R, TGCTATGCAGATGCGTTTGC					
Chromosomal region 1 (9 bp) (nucleotide positions 2678040 to 2678048)	BHZ526-1F, AGGAAATAGTTGGGGAGAGGT; BHZ5276-1R, AGCAAGATGATGGTTATGCTGT					
Chromosomal region 1 (20 bp) (nucleotide positions 448627 to 448782)	Bh743-7-1F, AGTACCTTTTCCAGCAGCAAG; Bh743-7-1R, GCAGAGGTGAAGCCGCTAAA					
Chromosomal region 2 (7 bp) (nucleotide positions 1283746 to 1283752)	BHZ755-2F, TGAGCATAAGCACGGCATTT; BHZ755-2R, ACTAATGCTGTATCTCCAATCCA					
Final ONT-Illumina hybrid assembly annotation^*f*^						
Total no. of coding sequences	2,652	2,590	2,588		2,595	
No. of coding genes	2,623	2,557	2,558		2,566	
Total no. of rRNAs	40	40	40		40	
No. of pseudogenes	29	33	30		29	
Accession numbers^*g*^						
SRA database						
ONT	SRR10609650	SRR10609648	SRR10609646		SRR10609644	
Illumina	SRR10609649	SRR10609647	SRR10609645		SRR10609643	
PacBio	SRR15444232	SRR15444231	SRR15444230		SRR15444229	
GenBank nucleotide database						
Chromosome (ONT-Illumina hybrid assembly)	CP046932	CP046930	CP046928		CP046926	
Plasmid (ONT-Illumina hybrid assembly)	CP046933	CP046931	CP046929		CP046927	

a Obtained with NanoPack (Nanostat) and LongQC.

b Obtained with LongQC. NA, not applicable.

c Obtained with FastQC.

d Obtained with QUAST and Illumina short-read remapping by running BBMap in Geneious.

e Numbers of regions and nucleotides that were manually inserted per chromosome in each genome after PCR and Sanger sequencing confirmation. Chromosomal region 1 of the BHZ755 genome was corrected using primers Bh743-7-1F and Bh743-7-1R.

f Obtained from the NCBI PGAP annotation files.

g Accession numbers corresponding to the sequencing runs deposited in the SRA database and accession numbers of the nucleotide sequences deposited in the GenBank sequence database.

The hybrid approach applied here allowed reconstruction of high-quality genomes (in terms of completeness and accuracy). These genomes contribute to expanding the catalogue of B. hyodysenteriae genomes and will serve as a basis for molecular epidemiology studies of swine dysentery.

### Data availability.

Annotated genomes have been deposited in GenBank under the BioProject accession number PRJNA594292 and BioSample accession numbers SAMN13511716, SAMN13511717, SAMN13511718, and SAMN13511719, and their accession numbers are listed in Table 1. ONT and Illumina raw data sets are archived in the SRA database (Table 1). Demultiplexed PacBio raw data (BioProject accession number PRJNA754405) are also archived in the SRA database (Table 1).

## References

[1] Speiser S. 2008. Untersuchungen zu *Lawsonia intracellularis*, *Brachyspira hyodysenteriae* und *Brachyspira pilosicoli* Infektionen bei Absetz und Mastschweinen in der Schweiz. Dr. med. vet. dissertation. Vetsuisse Faculty, University of Bern, Bern, Switzerland.

[2] Kirchgässner C, Schmitt S, Borgström A, Wittenbrink MM. 2016. Antimicrobial susceptibility of *Brachyspira hyodysenteriae* in Switzerland. Schweiz Arch Tierheilkd 158:405–410. doi:10.17236/sat00066. 27504836

[3] García-Martín AB, Perreten V, Rossano A, Schmitt S, Nathues H, Zeeh F. 2018. Predominance of a macrolide-lincosamide-resistant *Brachyspira hyodysenteriae* of sequence type 196 in Swiss pig herds. Vet Microbiol 226:97–102. doi:10.1016/j.vetmic.2018.10.007. 30389050

[4] De Coster W, D'Hert S, Schultz DT, Cruts M, Van Broeckhoven C. 2018. NanoPack: visualizing and processing long-read sequencing data. Bioinformatics 34:2666–2669. doi:10.1093/bioinformatics/bty149. 29547981 PMC6061794

[5] Martin M. 2011. Cutadapt removes adapter sequences from high-throughput sequencing reads. EMBnet J 17:10–12. doi:10.14806/ej.17.1.200.

[6] Tapia-Paniagua ST, Chabrillon M, Díaz-Rosales P, de la Banda IG, Lobo C, Balebona MC, Morinigo MA. 2010. Intestinal microbiota diversity of the flat fish *Solea senegalensis* (Kaup, 1858) following probiotic administration. Microb Ecol 60:310–319. doi:10.1007/s00248-010-9680-z. 20556376

[7] Fukasawa Y, Ermini L, Wang H, Carty K, Cheung MS. 2020. LongQC: a quality control tool for third generation sequencing long read data. G3 (Bethesda) 10:1193–1196. doi:10.1534/g3.119.400864. 32041730 PMC7144081

[8] Andrews S. 2010. FastQC: a quality control tool for high throughput sequence data. Babraham Bioinformatics, Cambridge, United Kingdom. https://www.bioinformatics.babraham.ac.uk/projects/fastqc.

[9] Bolger AM, Lohse M, Usadel B. 2014. Trimmomatic: a flexible trimmer for Illumina sequence data. Bioinformatics 30:2114–2120. doi:10.1093/bioinformatics/btu170. 24695404 PMC4103590

[10] Wick RR, Judd LM, Gorrie CL, Holt KE. 2017. Unicycler: resolving bacterial genome assemblies from short and long sequencing reads. PLoS Comput Biol 13:e1005595. doi:10.1371/journal.pcbi.1005595. 28594827 PMC5481147

[11] Wick RR, Schultz MB, Zobel J, Holt KE. 2015. Bandage: interactive visualization of *de novo* genome assemblies. Bioinformatics 31:3350–3352. doi:10.1093/bioinformatics/btv383. 26099265 PMC4595904

[12] Gurevich A, Saveliev V, Vyahhi N, Tesler G. 2013. QUAST: quality assessment tool for genome assemblies. Bioinformatics 29:1072–1075. doi:10.1093/bioinformatics/btt086. 23422339 PMC3624806

[13] Bushnell B. 2014. BBMap: a fast, accurate, splice-aware aligner. Lawrence Berkeley National Laboratory, Berkeley, CA.

[14] Tatusova T, DiCuccio M, Badretdin A, Chetvernin V, Nawrocki EP, Zaslavsky L, Lomsadze A, Pruitt KD, Borodovsky M, Ostell J. 2016. NCBI Prokaryotic Genome Annotation Pipeline. Nucleic Acids Res 44:6614–6624. doi:10.1093/nar/gkw569. 27342282 PMC5001611

